# Leucine-rich α2-glycoprotein-1 upregulation in plasma and kidney of patients with lupus nephritis

**DOI:** 10.1186/s12882-020-01782-0

**Published:** 2020-04-06

**Authors:** Yi Yang, Ran Luo, Yichun Cheng, Tingting Liu, Wei Dai, Yueqiang Li, Shuwang Ge, Gang Xu

**Affiliations:** Department of Nephrology, Tongji Hospital Affiliated with Tongji Medical College, Huazhong University of Science and Technology, 1095 Jie Fang Avenue, Wuhan, Hubei 430030 People’s Republic of China

**Keywords:** Leucine-rich α2-glycoprotein-1, Lupus nephritis, Proliferation, Apoptosis, Inflammation

## Abstract

**Background:**

Increased leucine-rich α2-glycoprotein-1 (LRG1) has been observed in various inflammatory and autoimmune diseases. We aimed to explore the expression and role of LRG1 in lupus nephritis (LN).

**Methods:**

Plasma LRG1 (pLRG1) was measured by enzyme-linked immunosorbent assay in 101 patients with renal biopsy-proven LN and 21 healthy controls (HC). Relationships between pLRG1 and clinical and pathological characteristics were analyzed. The expression of LRG1 in peripheral blood leukocytes and kidney was detected by flow cytometry, immunohistochemistry and immunofluorescence, respectively. Further cell experiments were focused on the role of LRG1.

**Results:**

We found that LRG1 was expressed in plasma, some peripheral blood leukocytes, proximal tubule and several inflammatory cells. The levels of LRG1 in plasma, peripheral blood leukocytes and kidney were elevated in LN patients as compared to HC. Plasma expression levels of LRG1 correlated positively with renal function and renal disease activity, and reflect specific pathologic lesions in the kidneys of patients with LN. Interleukin-1β and interleukin-6, not tumor necrosis factor-α and interferon γ induced the LRG1 expression in human renal tubular epithelial cell line. Moreover, stimulation of recombinant human LRG1 could inhibit late apoptosis, promote proliferation and regulate expression of inflammatory factors and cytokines.

**Conclusions:**

Plasma expression levels of LRG1 were associated with renal function, disease activity, and pathology in LN. It might also be involved in renal inflammation, proliferation and apoptosis of endothelial cells. LRG1 might be a potential prognosis novel predictor in LN patients.

## Background

Systemic lupus erythematosus (SLE) is a complicated and multisystem autoimmune disease [1–3]. Lupus nephritis (LN) is a serious and common manifestation of SLE, and is characterized by autoantibody-mediated activation of inflammatory response in the kidney [2, 4, 5]. About 35% of patients show LN symptoms when SLE is diagnosed and 50–60% will develop LN during the first 10 years of disease [6, 7]. Up to 26% of LN patients develop end-stage renal disease [8, 9]. And LN is a major contributor to mortality of SLE despite recent advances [3]. Hence, early diagnosis and management of LN is of tremendous importance.

For LN, etiology and pathogenesis are incompletely understood. Currently, LN is gauged by proteinuria, urinary sediment, creatinine clearance, anti-double-standed DNA antibodies and complement component with confirmation by kidney biopsies. Due to the invasive nature of kidney biopsies and these traditional markers are not perfect in assessing whether active LN is present or not, and none of them can anticipate the course of LN. It is of significance to explore novel sensitive and specific biomarkers of LN.

Leucine-rich alpha-2-glycoprotein 1 (LRG1) is an approximately 50-kDa plasma glycoprotein isolated from human serum in 1977 [10]. It contains 23% carbohydrate by weight and it consists of 312 amino acid residues, 66 of which are leucines. LRG1 has been reported to be expressed by the endothelial cells, neutrophils, macrophages and liver cells [11–13]. The precise function of LRG1 remains unknown, but accumulating evidences showed that LRG1 is closely correlated with various types of cancers, such as lung, oral, ovarian, gastrict, pancreatic and biliary tract cancers [14–18]. Moreover, LRG1 is identified as an inflammatory protein in human serum and highly expressed in various kinds of benign inflammatory and autoimmune diseases, including suspected acute appendicitis of children [19], activated ulcerative colitis [12], rheumatoid arthritis [20, 21], adult-onset Still’s disease [22] and asthma [23]. Especially, Ahn et al. found serum LRG, namely LRG1, is elevated in patients with SLE and correlates with disease activity [24].

We supposed that LRG1 was closely correlated with LN with the following evidences. Firstly, LN is a typical inflammatory and autoimmune disease, and LRG is elevated in patients with SLE [24]. Secondly, for kidney diseases, increased levels of LRG1 was identified in chronic kidney disease [25], sepsis patients with acute kidney injury [26], classical galactosemic patients with subclinical kidney insufficiency [27], children with idiopathic nephrotic syndrome [28] by using a plasma/serum/urine proteomics approach, but the proteomics studies just revealed a hint that LRG1 might be associated with kidney diseases. Thirdly, a mouse model of renal tubular injury indicated that LRG1 was a potential urinary biomarker [29]. Finally, Wang et al. reported that LRG1 promoted angiogenesis in endothelial cells [30]. Angiogenesis widely exists in kidney of LN. Therefore, studying roles of LRG1 in LN is a rich research area.

Thus, we conducted a study to determine positioning and compare the expression of LRG1 levels in plasma, peripheral blood leukocyte and kidney tissue in LN patients and healthy controls, to determine whether plasma LRG1 (pLRG1) was a biomarker for disease activity of LN and to explore the possible mechanisms.

## Methods

### Patients and specimens

A total of 101 biopsy-proven LN patients, 21 healthy controls (HC) subjects were recruited for this study. All of the patients were biopsy-proven LN according to the 2003 International Society of Nephrology (ISN)/Renal Pathology Society (RPS) classification criteria for LN. For the included patients, baseline clinical data at the time of renal biopsy were recorded. Venous plasma samples of LN patients at the time of renal biopsy and HC subjects were obtained and stored at − 80 °C for batch analysis. Informed consent was obtained from these participants. The protocol was approved by the Ethical Committee of Tongji Hospital, which is affiliated with Tongji Medical College, Huazhong University of Science and Technology (Project No. TJ-IRB20181106).

### LRG1 enzyme-linked immunosorbent assay (ELISA)

Concentrations of pLRG1 were detected using a human ELISA kit (ImmunoBiological Laboratories Co Ltd., Takasaki, Gunma, Japan) according to the manufacturer’s protocol.

### Flow cytometric analysis of LRG1 expression in peripheral blood leukocytes from LN patients and healthy controls

To detect LRG1 expression on neutrophils (CD15+), monocytes (CD14+), NK cells (CD3- CD56+), T cells (CD3+), B cells (CD19+) in peripheral blood leukocytes, we used the following antibodies from BioLegend: phycoerythrin-conjugated (PE-conjugated) anti–human CD15 Ab; PerCP/ Cy5.5-conjugated anti–human CD14 Ab; allophycocyanin-conjugated (APC-conjugated) anti–human CD3 Ab; BV421-conjugated anti–human CD56 Ab; PE-conjugated anti–human CD19 Ab. In addition, we used FITC-conjugated anti–human LRG1 Ab from ASSAYPRO. Flow cytometry measurements were performed using a FACS Calibur flow cytometer (Becton Dickinson, LSRII).

### Immunohistochemistry (IHC)

IHC was conducted using the UltraVision Quanto Detection System HRP DAB kit (Gene Tech). Kidney tissues were deparaffinized and rehydrated. For antigen retrieval, sections were submerged in citrate buffer (pH 6.0) at 100 °C for 30 min. After blocking endogenous peroxidase activity and nonreactive sites, primary rabbit anti-human LRG1 antibody (ATLAS; 1:200) were incubated overnight at 4 °C. Goat-anti rabbit immunoglobulins and diaminobenzidine tetrahydrochloride solution were used to detect antibody binding.

### Immunofluorescence staining and co-localization assay

The paraffin sections were de-paraffinized, antigen retrieval and incubated in blocking serum according to the same method as IHC. After incubation with the primary antibody (rabbit anti-LRG1, ATLAS, dilution 1:50; fluorescein *Lotus tetragonolobus* lectin, Vector Laboratories, dilution 1:100; biotinylated *Dolichos biflorus* agglutinin, Vector Laboratories, dilution 1:250; rabbit anti-thiazide-sensitive Nacl cotransporter, Millipore, dilution 1:500) diluted in PBS at 4 °C overnight, the slides were incubated with the corresponding secondary antibody. For double staining, cells were incubated with the mixture of two primary antibodies (1:50 rabbit anti-LRG1 and 1:25 mouse anti-CD68, CD3, CD11c and CD19) and a mixture of the two secondary antibodies. Finally, all sections were counterstained with DAPI and sealed with 50% buffered glycerol.

### Cell line and culture

The human renal tubular epithelial cell line HK-2 and Human umbilical vein endothelial cell line HUVEC, were obtained from the Cell Bank of the Chinese Academy of Sciences (Beijing, China). Cells were maintained in RPMI-1640 medium supplemented with 10% fetal bovine serum and 100 U/mL penicillin streptomycin and maintained at 37 °C in a humidified atmosphere containing 5% CO2.

### Western blot

Total cell lysates from HK-2 cell lines were extracted with radioimmunoprecipitation (RIPA) assay buffer containing phenylmethane sulfonyl fluoride (PMSF) and protease inhibitors. Primary antibodies against LRG1 (13224–1-AP, Proteintech, 1:1500) and β-Actin or β-Tublin (1:2000) and secondary antibody horseradish peroxidase were used.

### Evaluation of cell apoptosis

Cell apoptosis was probed using the FITC Annexin V/PI Apoptosis Detection Kit (AntGene, Wuhan, China) according to the manufacturer’s instructions. Evaluation of cell proliferation. The cell counting kit-8 (CCK-8) assay (Promoter, Wuhan, China) was used to evaluate cell proliferation. The absorbance was measured in a microplate reader at 450 nm.

### RNA extraction and quantitative real-time PCR (qRT-PCR) analysis

Total RNA was isolated from cells by the TRIzol reagent (Invitrogen) and was reversely transcribed into cDNA using PrimeScript RT Reagent (Takara, Japan) after RNA quantification. qRT-PCR, was performed on an Applied Biosystems 7500 Real-time PCR System using SYBR Premix Ex Taq Kit (TaKaRa, Dalian, China) following the manufacturer’s protocols. All primer sequences were showed in Additional file 1: Table S1.

### Statistical analysis

To show the normal distribution of variables, the Kolmogorov-Smirnov test was used by SPSS 23.0 software. GraphPad Prism 6 and were used for other statistical analysis and visualization. The t-test was used for univariate parametric comparisons. Mann Whitney U test or Kruskal-Wallis test followed by Dunn’s multiple comparison test were used for univariate nonparametric comparisons. Spearman’s correlation analyses were performed to evaluate the association between nonparametric variables. A two-tailed *P* < 0.05 was considered statistically significant.

## Results

### Plasma concentrations of LRG1 in HC and patients with LN

**Fig. 1:**
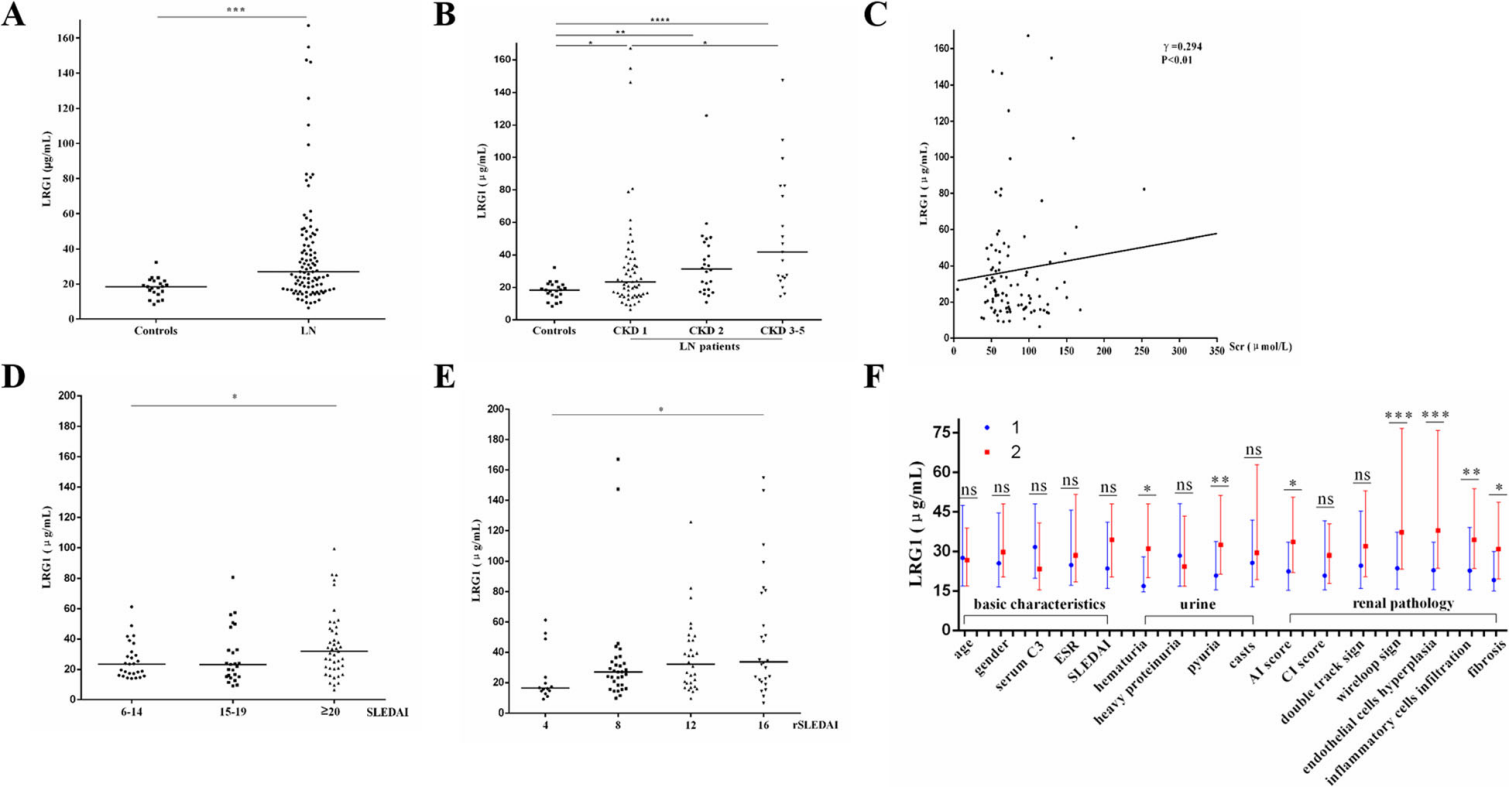
Plasma leucine-rich alpha-2 glycoprotein 1 (LRG1) levels in lupus nephritis (LN) patients and its correlation to indicators. **a** Plasma LRG1 levels in LN patients and healthy controls. **b** Differences in plasma concentrations of LRG1 between different patients with different CKD grades. **c** Plasma levels of LRG1 are positively correlated with serum creatinine (γ = 0.294, *P* < 0.01). **d**, **e** Differences in plasma concentrations of LRG1 between different patients with different SLE activity grade (SLEDAI) and renal SLEDAI (rSLEDAI). **f** Differences in plasma concentrations of LRG1 between different patients with other different clinical and pathological indicators, 1 and 2 are representative < and ≥ median: 29.0 years for age, 5.0 AI score, 4.0 CI score, 0.4 for complement 3 (C3); female and male for gender; < and ≥ 20 for SLEDAI;<20 and ≥ 20 mm/h for erythrocyte sedimentation rate (ESR); − and + for hematuria, heavy proteinuria, pyuria, casts, double track sign, wireloop sign, endothelial cells hyperplasia and fibrosis; light and heavy for inflammatory cell infiltration. Symbols represent individual data points with the median as a horizontal line in Fig. 1a, b, d and e. Data are presented as bar graphs with the median and 25–75th percentile of the plasma LRG1 concentrations in Fig. 1f (ns, not significant; *P < 0.05; **P < 0.01; ***P < 0.001; ****P < 0.0001)

For the included 101 LN patients and 21 HC, there was no statistically significant difference in the age or the sex ratio between the two groups (Table 1). pLRG1 levels were significantly elevated in patients with LN compared with those in the HC (*P* < 0.001; Fig. 1a).

**Table 1:** Demographics and clinical characteristics of patients with lupus nephritis and healthy controls

Parameters	lupus nephritis	healthy controls	***P*** value
n	101	21	–
Female, n (%)	82 (81.2%)	14 (66.7%)	0.14^a^
Age, median (IQR), years	29.0 (24.0–38.0)	31.0 (27.5–39.5)	0.15^b^
Mean arterial pressure, mean (SEM), mmHg	97.8 (1.4)	91.7 (1.5)	< 0.01^c^
LN duration, median (IQR), years	0.3 (0.1–1.4)	–	–
SLEDAI-2 k, mean (SEM)	17.8 (0.6)	–	–
Renal SLEDAI, median (IQR)	12.0 (8.0–16.0)	–	–
AI score, median (IQR)	5.0 (4.0–7.0)	–	–
CI score, median (IQR)	4.0 (3.0–5.0)	–	–
24-h urine protein, median (IQR), g	2.5 (1.0–7.0)	–	–
Hemoglobin, mean (SEM), g/L	108.5 (2.0)	–	–
Serum albumin, mean (SEM), g/L	26.9 (0.8)	–	
Serum creatinine, median (IQR), μmol/L	70.0 (56.0–99.8)	–	–
Blood urea nitrogen, median (IQR), mmol/L	6.3 (4.5–8.2)	–	–
Uric acid, median (IQR), μmol/L	342.5 (273.5–418.8)	–	–
Blood phosphorus, median (IQR), mmol/L	1.2 (1.1–1.4)	–	–
Complement 3, median (IQR), g/L	0.4 (0.3–0.6)	–	–
Complement 4, median (IQR), g/L	0.07 (0.04–0.1)	–	–

Values are expressed as mean (SEM), median (25–75th percentile) or n (%). ^a^Chi-square test. ^b^Mann Whitney U test. ^c^ t-test.

SLEDAI-2 k, SLE Disease Activity Index 2000; IQR, interquartile range; AI, activity index; CI, chronicity index

### Plasma concentrations of LRG1 in patients with LN according to stages of CKD

All LN patients were divided into 3 groups according to stages of CKD: stage 1 (*n* = 58), stage 2 (*n* = 24) and stage 3–5 (*n* = 19) of CKD. pLRG1 levels were higher in all stage 1, stage 2 and stage 3–5 groups than in HC group (*P* < 0.05, *P* < 0.01 and *P* < 0.0001, respectively; Fig. 1b). pLRG1 levels were not significantly different between stage 1 group and stage 2 groups (*P* = 0.147; Fig. 1b). However, pLRG1 levels in stage 3–5 of CKD significantly differed from the stage 1 (*P* < 0.05; Fig. 1b). pLRG1 levels in stage 3–5 of CKD was not significantly higher than in the stage 2 (*P* = 0.121; Fig. 1b). And the Spearman’s correlation analyses showed that pLRG1 level was weakly positively correlated to serum creatinine (γ = 0.294; *P* < 0.01; Fig. 1c). All these showed that pLRG1 level had a tendency to increase with stage of CKD.

### Plasma concentrations of LRG1 in patients with LN according to disease activity status

All patients’ Systemic Lupus Erythematosus Disease Activity Index (SLEDAI) and renal Systemic Lupus Erythematosus Disease Activity Index (rSLEDAI) scores were calculated to evaluate overall and renal disease activity [31]. All LN patients were divided into a low active group (6–14 scores; *n* = 29), a moderate active group (15–19 scores; *n* = 27) or a high active group (≥ 20 scores; *n* = 45) according to their SLEDAI scores. pLRG1 levels were higher in score ≥ 20 group than in score 6–14 group (*P* = 0.043; Fig. 1d).

The rSLEDAI scores were a sum of four urinary items including hematuria (≥ 5 red blood cells/high-power field), proteinuria (≥ 0.5 g/24 h or spot urine protein/Cr ratio > 0.5), pyuria (≥ 5 white blood cells/high-power field) and casts (composed of heme, granule, or red blood cells). The scale of each parameter was 0 or 4, and total rSLEDAI score were 0, 4, 8, 12, or 16 [31]. pLRG1 levels were higher in score 16 group than in score 4 group (*P* = 0.019; Fig. 1e).

### Plasma concentrations of LRG1 in patients with LN according to other clinical and pathological characteristics

All LN patients were respectively divided into number 1 group and number 2 group according to age (< and ≥ median: 29.0 years), gender (female and male), SLEDAI (< and ≥ 20), hematuria (− and ≥ +), heavy proteinuria (− and +), pyuria (− and +), casts (− and +), Activity index (AI) score (< and ≥ median: 5), Chronicity index (CI) score (< and ≥ median: 4.0), serum complement 3 (< and ≥ median: 0.4), ESR (< and ≥ 20), double track sign (− and +), wireloop sign (− and +), endothelial cells hyperplasia (− and +), inflammatory cells infiltration (light and heavy) and fibrosis (− and +). pLRG1 levels were higher in several number 2 groups than in number 1 groups for hematuria, pyuria, AI score, wireloop sign, endothelial cells hyperplasia, inflammatory cells infiltration, and fibrosis (all *P* < 0.05; Fig. 1f; Additional file 2: Table S2).

### LRG1 expression in peripheral blood leukocytes

**Fig. 2:**
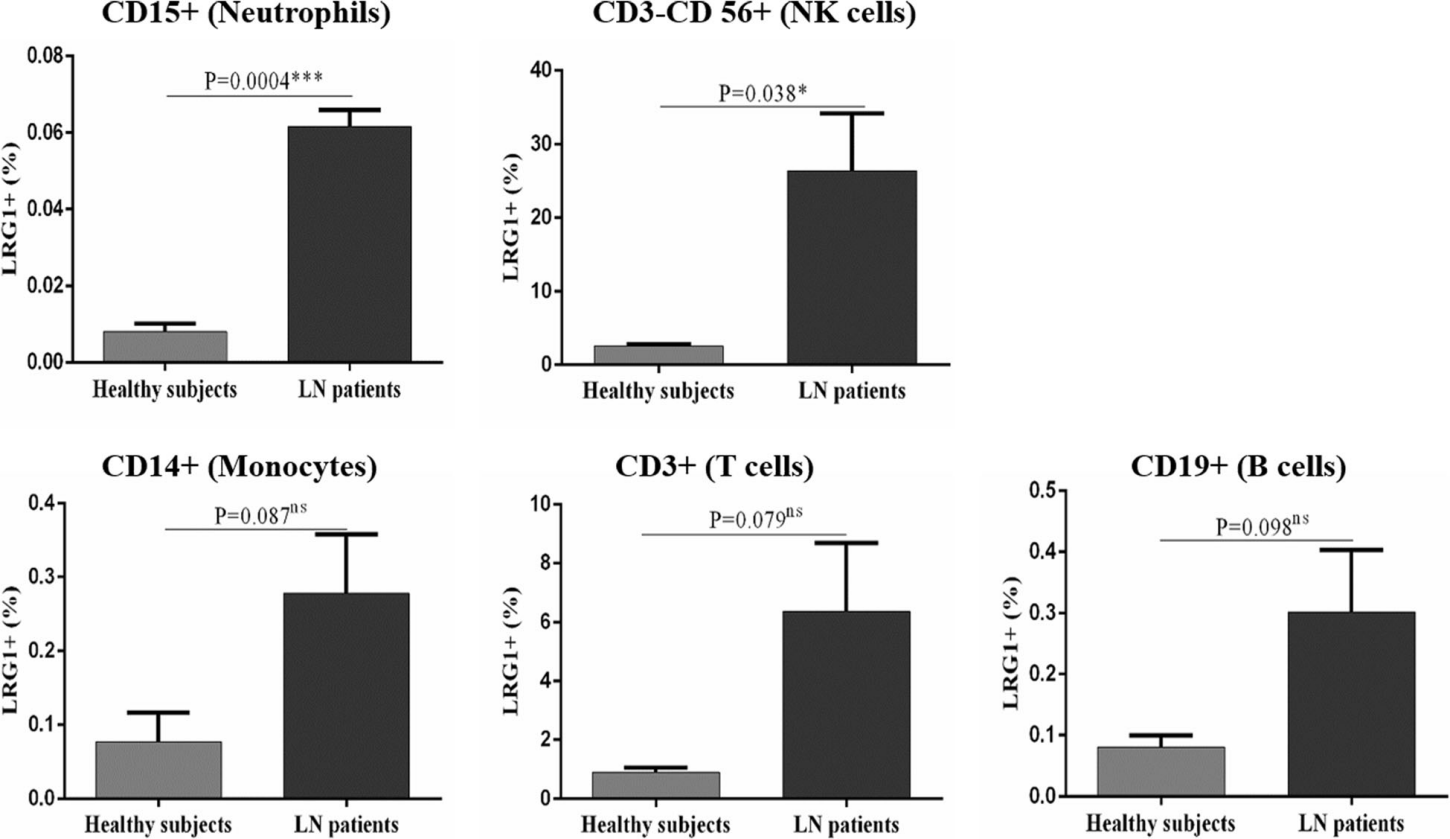
Flow cytometric analysis of leucine-rich alpha-2 glycoprotein 1 (LRG1) expression in peripheral blood leukocytes of LN patients and HC. Quantitative presentations of LRG1+ cells in CD15+ (Neutrophils), CD3-CD 56+ (NK cells), CD14+ (Monocytes), CD3+ (T cells) and CD19+ (B cells) cells for healthy controls and lupus nephritis (LN) patients. *n* = 3 per group. ns, not significant; *P < 0.05; ***P < 0.001

To identify the potential sources of LRG1 production, we investigated LRG1 expression in peripheral blood leukocytes (Fig. 2). The result showed that the percentages of LRG1+ cells present in CD15+ and CD3-CD56+ cell populations were higher in patients with LN when compared with HC subjects (*P* = 0.0004 and *P* = 0.04, respectively), but not in CD14+ (*P* = 0.09), CD3+ (*P* = 0.079) and CD19+ (*P* = 0.098) cell populations. Moreover, large proportions of CD3-CD56+ and CD3+ expressed LRG1 but the percentages of LRG1+ cells in CD15+, CD14+ and CD19+ cell populations were very low (< 1%) both in LN patients and HC.

### Renal expression of LRG1 was higher in patients with LN

**Fig. 3:**
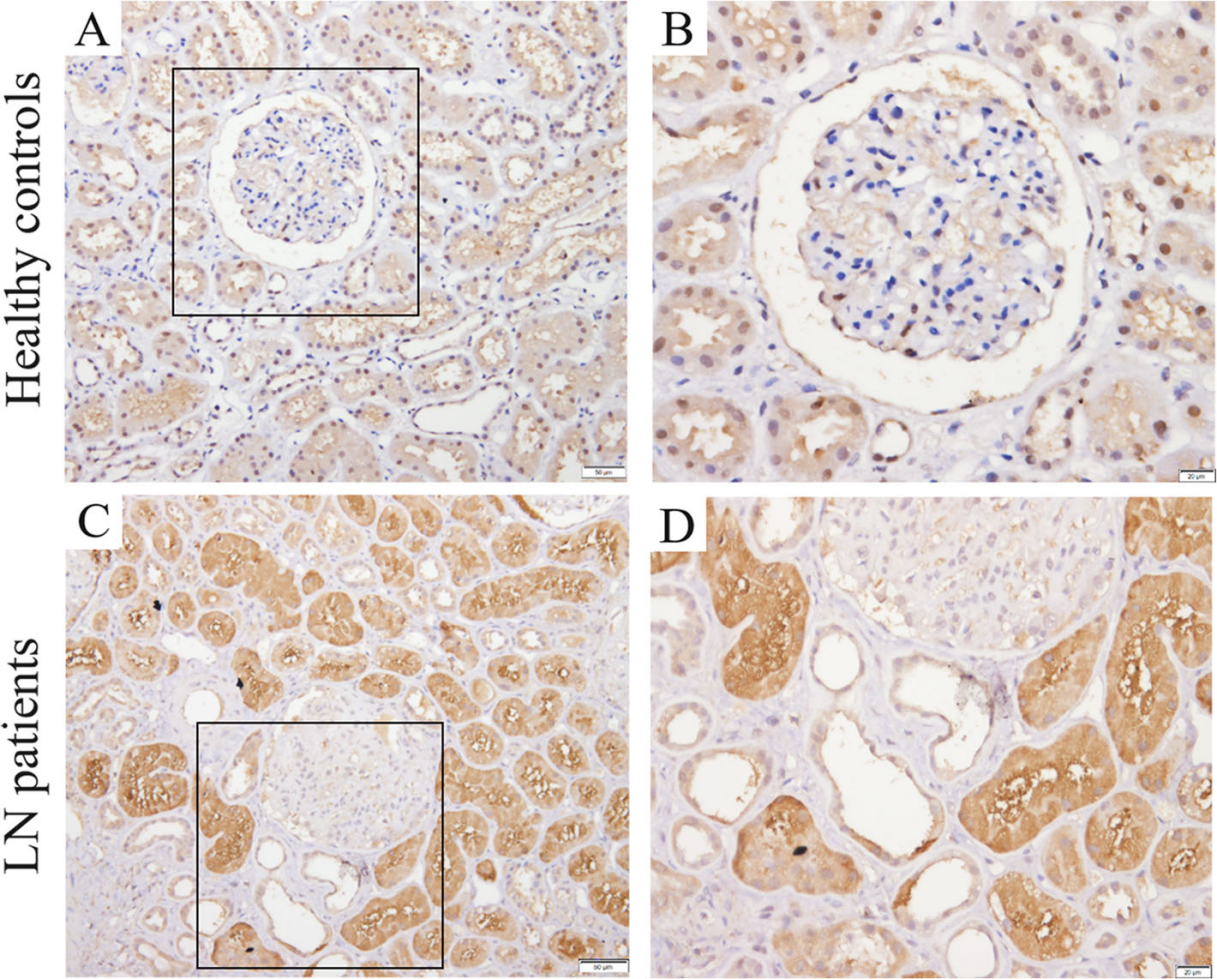
Renal cortical expression of leucine-rich alpha-2 glycoprotein 1 (LRG1) in human kidney biopsies. Representative photomicrographs of LRG1 staining in human renal cortical tissue from normal subjects (**a** and **b**), lupus nephritis (LN) patients (**c** and **d**). Hematoxylin stain; original magnification, × 200 (A and C, Scale bars, 50 μm); × 400 (**b** and **d**, Scale bars, 20 μm)

LRG1 expression in the LN patients and HC was measured by using immunohistochemistry. As shown, LRG1 was mainly expressed at some tubes; at renal interstitial and glomerulus, there was only dotted expression of LRG1. LRG1 expression in the LN patients group was significantly higher than in the HC group (*P* < 0.01; Fig. 3 and Additional file 3: Figure S1).

### LRG1 was located at proximal tubule and some inflammatory cells in kidney

**Fig. 4:**
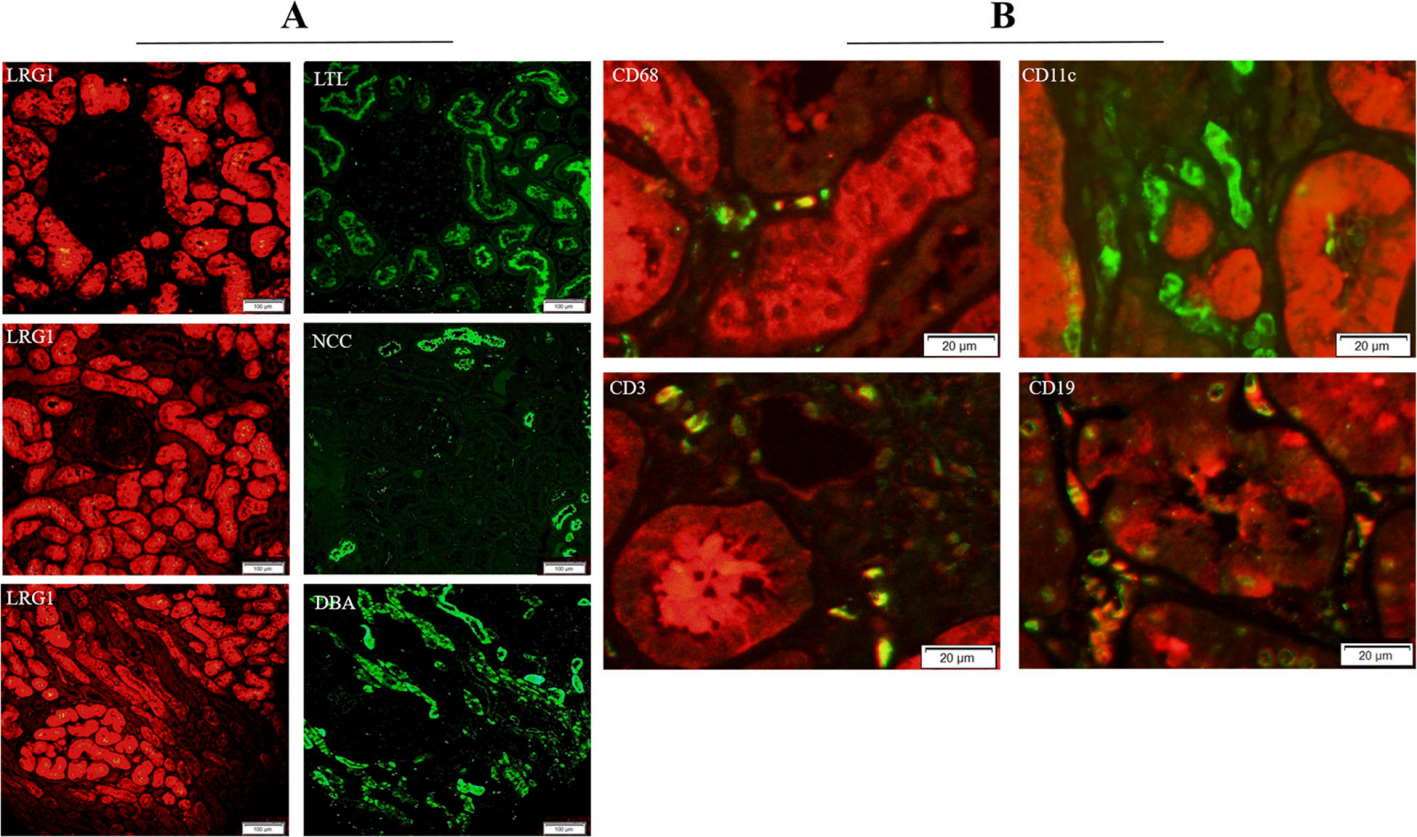
Leucine-rich alpha-2 glycoprotein 1 (LRG1) was expressed at proximal tubules and several inflammatory cells in kidneys of lupus nephritis (LN) patients. **a** Continuous kidney paraffin sections of LN patients were co-stained with anti-LRG1 and anti- *Lotus tetragonolobus* lectin (LTL)/Nacl cotransporter (NCC)/*Dolichos biflorus* agglutinin (DBA) antibodies. Representative images of the border areas are shown. Scale bars, 100 μm. **b** Paraffin sections of LN patients were co-stained with anti-LRG1 and anti-68/11c/3/19 antibodies. Representative images of the border areas are shown. Scale bars, 20 μm

It was shown that LRG1 was expressed at proximal tubule (marker: *Lotus tetragonolobus* lectin, LTL) but not at distal tubule (marker: Nacl cotransporter, NCC) and collection tube (marker: *Dolichos biflorus* agglutinin, DBA) by immunofluorescence staining for continuous kidney section (Fig. 4a). Immunofluorescence co-localization staining showed that cells of CD68+ (macrophages), CD3+ (T cells) and CD19+ (B cells) co-dyed with LRG1 and cells of CD11c + (dendritic cells) did not co-dye with LRG1 (Fig. 4b).

### IL-1β and IL-6 induced HK-2 cells to produce LRG1

**Fig. 5:**
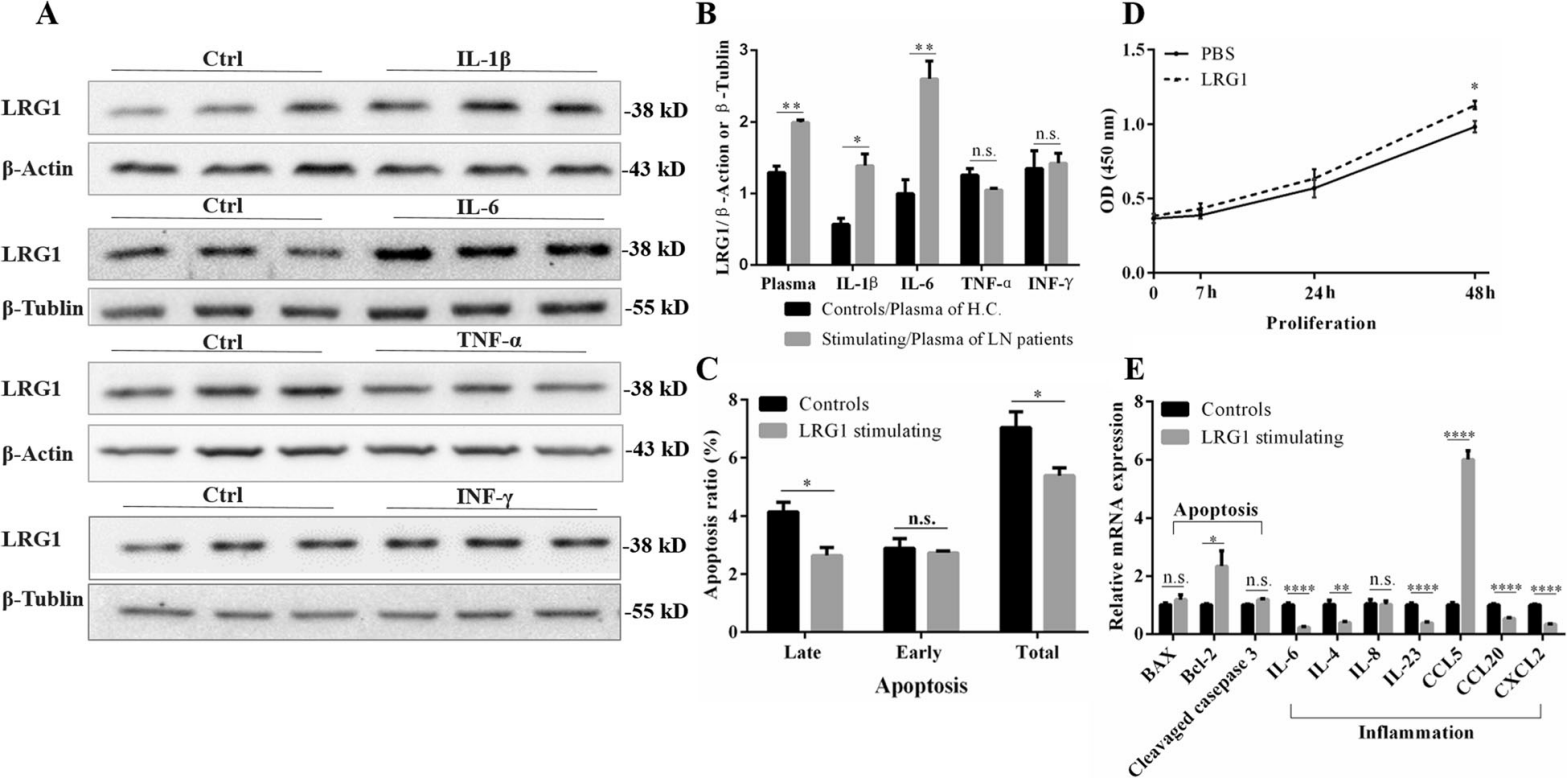
Leucine-rich alpha-2 glycoprotein 1 (LRG1) could be induced by proinflammatory cytokines in HK-2 cell line and its effects on HUVEC cell line. **a**, **b** The expressions of LRG1 were analysed by western blot in HK-2 cells stimulated with 20 ng/mL IL-1β, IL-6, TNF-α and INF-γ for 8 h (n = 3). Analyses were performed by Student’s t test. **c** HUVEC cells stimulated by 500 ng/mL rhLRG1 for 8 h were double stained with FITC-conjugated anti-Annexin Vantibody and PI, followed by flowcytometry analysis for cell apoptosis. **d** CCK-8 assay was performed to assess cell proliferation in HUVEC cell line. **e** HUVEC cell line was stimulated by 500 ng/mL rhLRG1 for 24 h. The expression of mRNA was analyzed by real-time RT-PCR and GAPDH was used as an internal control for grayscale analysis. Data are shown as mean ± SEM. **c**-**e** analyses were controls groups vs. 500 ng/mL LRG1 stimulating groups performed by Student’s t test. n.s., not significant. *P < 0.05, **P < 0.01, ***P < 0.001, ****P < 0.0001. LRG1: leucine-rich α2-glycoprotein 1; IL: Interleukin; TNF: Tumor necrosis factor; INF: Interferon; CCL: chemokine (C-C motif) ligand; CXCL: Chemokine (C-X-C Motif) Ligand 1

To determine whether the inflammatory factors could induce the production of LRG1, we stimulated HK-2 cells by IL-1β, IL-6, TNF-α and IFN-γ for 8 h. Western blot showed IL-1β and IL-6 could induce expression of LRG1 in HK-2 cells (Fig. 5a, b).

### Recombinant human LRG1 (rhLRG1) stimulating induced proliferation, inhibited late apoptosis, and regulated inflammation in HUVEC cells

Endothelial cell proliferation was one of the most vital pathological changes of LN. 500 ng/mL rhLRG1 was used to stimulate HUVEC cell line. The cell growth curves of 24 and 48 h, as assessed by the CCK-8 method, indicated that the growth rate was higher in the rhLRG1 stimulating group than control group (Fig. 5d). As shown in Fig. 5c, the late apoptotic rate was decreased, but the early apoptotic rate was not significantly different in the LRG1 stimulating (8 h) group compared with control group (Fig. 5c). Besides, LRG1 induced mRNA expression of Bcl-2 (Fig. 5e). These results indicated that LRG1 exerted more prominent pro-proliferative and anti-apoptotic effects on the HUVECs. Stimulation of rhLRG1 on HUVEC decreased the mRNA expression levels of IL-6, IL-4, IL-23, CCL20 and CXCL2, and increased that of CCL5 (Fig. 5e). It might be a multiple regulatory factor in inflammation.

## Discussion

This is the first LRG1 study in the population of biopsy-proven LN so far. In this study, we found that in patients with LN, LRG1 was expressed in plasma, several white blood cells of peripheral blood, at proximal tubule and several inflammatory cells of kidney. And we also examined its potential effects on kidney.

In plasma, level of LRG1 might be a good indicator of renal function and renal disease activity of LN. pLRG1 was positively correlated with serum creatinine levels and stages of CKD. It was reported that high level of serum LRG1 pointed to a subclinical kidney insufficiency in classical galactosemic patients by proteomics methods [27]. Furthermore, fluctuations of pLRG1 levels were found to reflect renal function in LN patients, thus potentially serving as a helpful biomarker in the clinical follow-up. Meanwhile, pLRG1 was a biomarker of renal disease activity of LN. In patients with highest rSLEDAI score, level of pLRG1 was higher than that in lowest rSLEDAI score. LRG1 was reported to be involved in inflammatory and autoimmune diseases. In active LN, the inflammatory and autoimmune reactions were more serious. In previous study, serum LRG1 concentrations correlated with disease activity in SLE, rheumatoid arthritis, Crohn’s disease, ulcerative colitis and adult-onset Still’s disease [12, 20, 22, 24, 32].

It was reported that serum LRG1 was elevated in patients with SLE and correlated with disease activity [24]. Similarly, we found LN patients with higher SLEDAI had higher pLRG1 concentrations. Differently, in our study, we included biopsy-proven LN patients instead of SLE patients. We found pLRG1 was elevated in LN patients compared to HC and might be a good indicator of renal function and renal disease activity of LN.

One of the strengths of our study was the examination of pLRG1’s ability to predict renal pathology. Higher levels of pLRG1 existed in patients who had wireloop sign, heavy inflammatory cells infiltration and fibrosis in the kidneys. Wireloop sign characterized by a large number of immunocomplex deposited under the endothelium, and heavy infiltration of inflammatory cells indicated severe inflammatory and immune reactions in kidney, which was consistent with the association between pLRG1 and rSLEDAI above. Fibrosis in renal tubular interstitium reflected chronic injury and loss of function of kidney, which was also consistent with the association between pLRG1 and renal function above. Recently, Lee et al. revealed that urinary LRG1 was a potential biomarker for renal tubular injury in mouse albumin overload model [29]. Therefore, LRG1 might be associated with renal inflammation, immunity, fibrosis and injury.

In addition, we revealed that pLRG1 elevated in patients with hyperplasia of renal endothelial cells. And the stimulation of rhLRG1 induced proliferation of HUVEC cell line. This might result from the following two possible reasons. On the one hand, LRG1 might be involved in renal inflammation and deposition of immune complexes, causing injury and further hyperplasia of endothelial cells. On the other hand, LRG1 was reported mitogenic to endothelial cells by modulating endothelial TGF-β signaling [30]. Hypercellularity of endothelial cells was one of basic pathological regenerations after injury in the glomeruli, which would further cause hematuria, proteinuria and decrease in glomerular filtration rate.

In our study, level of pLRG1 was elevated in LN patients compared with that in HC. The possible causes were as follows. First, we found in neutrophils and NK cells of peripheral blood, the LRG1 expression increased in LN patients compared to that in HC. Second, as a secretory protein, LRG1 was mainly expressed at proximal tubule. IHC showed the expression of LRG1 increased in kidney tubules of LN patients. Moreover, several inflammatory cells including macrophages, T cells and B cells of kidney could also expressed LRG1. So the secretion from kidney of LN patients might be increased.

In kidney, the expression of LRG1 also increased in LN patients. And the expression of LRG1 was positive in proximal tubule, and negative in distal tubule or collecting tube. Besides, some inflammatory cells infiltrated in glomerulus and renal interstitium, including macrophage, T cell and B cell, could also express LRG1. LRG1 could be induced by plasma of LN patients in HK-2 cell line. In order to determine whether proinflammatory cytokines might promote the production of LRG1, we chose IL-1β, IL-6, TNF-α and INF-γ to directly stimulate HK-2 cell line because LRG1 was expressed mainly at proximal tubule. It was reported that these four cytokines increased in LN and were important pathogenic factors. We found LRG1 could be induced by IL-1β and IL-6, but not TNF-α and INF-γ.

By combined with Figs. 2 and 3, we found in the fibrotic renal tissue, the expression of LRG1 was reduced. Because LRG1 was mainly and extensively expressed at the proximal tubule and proximal tubule was destroyed in the fibrotic tissue. However, several inflammatory cells, including macrophages, T cells and B cells, could also express LRG1. But, the total amount of LRG1 expression was reduced. Figure 1f showed that plasmic LRG1 level was higher in LN patients with renal fibrosis. The opposite conclusion might be explained by the following reasons: Firstly, Fig. 1f was the result of LRG1 level in plasma, but not kidney; Secondly, in the process of renal fibrosis, the destroyed proximal tubules might release LRG1 into the blood. However, the exact reason needs further studies.

Furthermore, we revealed that LRG1 might be associated with inflammation, proliferation and apoptosis of the endothelial cell. It could induce chemokine (C-C motif) ligand 5 (CCL5), but reduce CCL20, CXCL2, IL-4, IL-6 and IL-23 in HUVEC cell line. pLRG1 was elevated in patients with high renal disease activity and LRG1 could be induced by some inflammatory cytokines. Meanwhile, the stimulation of LRG1 could increase CCL5, but reduce some other cytokines. LRG1 might have bidirectional regulation of inflammation.

The reduced proinflammatory cytokines IL-6, IL-23, CCL20 and CXCL12 played antiinflammatory role in LN. IL-6 is a lymphokine produced by activated T cells and fibroblasts, which can make B cell precursors become antibody producing cells. In collaboration with colony stimulating factor, IL-6 can promote the growth and differentiation of original bone marrow-derived cells and enhance the lysis function of natural killer cells [33]. IL-23 is released from antigen presenting cells and induces expansion of Th17 cells and is necessary for their maintenance, thus forming the IL-23/IL-17 axis [34]. CCL20 is a chemokine of CC subfamily, which directly participates in the directional migration of dendritic cells and T cells through its ligand CCR6. CXCL12 is a classic inflammatory chemokine and can be secreted by a variety of cells, including lymphocytes, monocytes, and endothelial cells, to bind to its receptor, CXCR4. In glomerulonephritis, CXCR4 is overexpressed in parietal epithelial cells of the kidney, triggering their migration into the glomerular tuft where they form hyperplastic lesions [35]. LRG1 might participate in the inflammatory response by reducing these proinflammatory factors.

In HUVEC cell line, we found the stimulation of rhLRG1 not only promoted cell proliferation but also inhibited apoptosis, mainly late apoptosis. It was well known that inadequate apoptosis and excessive apoptosis were both harmful to LN, such as inadequate apoptosis of lymphocytes targeting autoantigens and excessive apoptosis of renal tubular cell. In fact, for endothelial cells, apoptosis and hyperplasia were both its reactions to injury. LRG1 inducing proliferation and reducing apoptosis of endothelial cells might together lead to proliferation of endothelial cells, which influenced renal structural and functional integrity. In this respect, LRG1 might be might be involved in pathological changes of kidney to injury.

However, pLRG1 did not correlate well with proteinuria, which is another important marker of LN severity. The possible reasons are as follows: Firstly, proteinuria is a relative early manifestation of LN, and renal insufficiency is a more serious manifestation of the disease progression. Proteinuria and renal function are not necessarily parallel, and their risk factors are not identical. Secondly, in our study, we found LRG1 had more effect on proliferation of endothelial cells, which might influence renal function. Proteinuria was effected more on damage to podocytes and charge barrier. However, the exact mechanism needs our further research on lupus and LRG1.

Our study has several limitations. First, the sample size of LN biopsies was relatively modest. Second, due to the cross-sectional design, the serial change in pLRG level according to the change in the progression or relief of LN and the association between pLRG1 and prognosis of LN could not be defined. Further large-sized, longitudinal studies were warranted.

## Conclusions

This work demonstrates that LRG1 are promising LN biomarkers. LRG1 was widely expressed in peripheral blood and kidney of LN. The level of plasma LRG1 was elevated in LN patients as compared to HC. Plasmic expression levels of LRG1 correlate positively with renal function and disease activity, and reflect specific pathologic lesions in the kidneys of patients with LN. It may also be involved in inflammation, proliferation and apoptosis of endothelial cells. Therefore, we suggest that this protein should be regularly checked in LN patients. However, this is the first LRG1 study in populations of LN, and further studies is needed to assess its clinical utility and functions of the disease.

## Supplementary information

**Additional file 1: Table S1.** Primers for quantitative real time reverse transcription polymerase chain reaction.

**Additional file 2: Table S2.** Differences in plasma concentrations of LRG1 between different patients with other different clinical and pathological indicators (The explanation of the Fig. 1f).

**Additional file 3: Figure S1.** LRG1 was stained in kidney of LN patients.

## Data Availability

The datasets used and analysed during the current study are available from the corresponding author on reasonable request.

## Abbreviations

LRG1: Leucine-rich α2-glycoprotein-1
LN: Lupus nephritis
pLRG1: plasma LRG1
HC: Healthy controls
SLE: Systemic lupus erythematosus
SLEDAI-2 k: SLE Disease Activity Index 2000
AI: Activity index
CI: Chronicity index
IL: Interleukin
TNF: Tumor necrosis factor
INF: Interferon
CCL: Chemokine (C-C motif) ligand
CXCL: Chemokine (C-X-C Motif) Ligand 1
