# Supplementary material for: Leucine-rich α2-glycoprotein-1 upregulation in plasma and kidney of patients with lupus nephritis
Source: BMC Nephrol. 2020 Apr 6;21:122. doi: 10.1186/s12882-020-01782-0 (PMC7137487; doi:10.1186/s12882-020-01782-0)
Supplement: Supplementary file 1 — Additional file 1: Table S1. Primers for quantitative real time reverse transcription polymerase chain reaction. [file 12882_2020_1782_MOESM1_ESM.docx]

**Table S1** Primers for quantitative real time reverse transcription polymerase chain reaction

|  | Forward (5’-3’) | Reverse (5’-3’) |
| --- | --- | --- |
| Human GAPDH | GGAAGCTTGTCAATGGAAATC | TGATGACCCTTTTGGCTCCC |
| Human BAX | TGACGGCAACTTCAACTGGG | GCACTCCCGCCACAAAGA |
| Human Bcl-2 | ATCGCCCTGTGGATGACTGA | GACAGCCAGGAGAAATCAAACAG |
| Human cleavage casepase-3 | ATCGCCCTGTGGATGACTGA | GACAGCCAGGAGAAATCAAACAG |
| Human IL-6 | ACTCACCTCTTCAGAACGAATTG | CCATCTTTGGAAGGTTCAGGTTG |
| Human IL-4 | GATGTCTGTTACGGTCAACTCG | ATGGGTCTCACCTCCCAACT |
| Human IL-8 | TTTTGCCAAGGAGTGCTAAAGA | AACCCTCTGCACCCAGTTTTC |
| Human IL-23 | AGTGTGGAGATGGCTGTGAC | AGGCTTGGAATCTGCTGAGT |
| Human CCL5 | CCAGCAGTCGTCTTTGTCAC | CTCTGGGTTGGCACACACTT |
| Human CCL20 | CAAGCAACTTTGACTGCTGTCTTGGA | TGCGCACACAGACAACTTTTTCTTTG |
| Human CXCL2 | CAACCCCGCATCGCCCAT | TGGCCTCTGCAGCTGTGTCTCTCT |
