# Supplementary material for: Leucine-rich α2-glycoprotein-1 upregulation in plasma and kidney of patients with lupus nephritis
Source: BMC Nephrol. 2020 Apr 6;21:122. doi: 10.1186/s12882-020-01782-0 (PMC7137487; doi:10.1186/s12882-020-01782-0)
Supplement: Supplementary file 2 — Additional file 2: Table S2. Differences in plasma concentrations of LRG1 between different patients with other different clinical and pathological indicators (The explanation of the Fig. 1f). [file 12882_2020_1782_MOESM2_ESM.docx]

**Table S2** Differences in plasma concentrations of LRG1 between different patients with other different clinical and pathological indicators (The explanation of the figure 1F)

| **Parameters** | **Number in Figure 1F** | **LRG1 (μg/mL)** | **^a^P** | |
| --- | --- | --- | --- | --- |
| **age** |  |  |  | |
| ＜29.0 years old | 1 | 27.6, 17.0-47.4 | 0.67 |  |
| ≥29.0 years old | 2 | 26.8, 16.9-38.8 |  | |
| **gender** |  |  |  | |
| female | 1 | 25.6, 16.5-44.6 | 0.43 | |
| male | 2 | 29.7, 20.3-47.9 |  | |
| **Complement 3** |  |  |  | |
| ＜0.4 g/L | 1 | 31.6, 19.9-47.9 | 0.11 | |
| ≥0.4 g/L | 2 | 23.4, 15.4-40.8 |  | |
| **ESR** |  |  |  | |
| ＜20 mm/h | 1 | 24.9, 17.2-45.7 | 0.58 | |
| ≥20 mm/h | 2 | 28.5, 18.4-51.6 |  | |
| **SLEADI** |  |  |  | |
| ＜20 | 1 | 23.6, 16.0-41.1 | 0.13 | |
| ≥20 | 2 | 34.3, 20.3-48.0 |  | |
| **hematuria** |  |  |  | |
| - (negative) | 1 | 16.9, 14.6-28.1 | 0.01* | |
| + (positive) | 2 | 31.0, 20.0-47.9 |  | |
| **heavy proteinuria** |  |  |  | |
| - | 1 | 28.5, 16.8-48.1 | 0.54 | |
| + | 2 | 24.3, 16.8-43.4 |  | |
| **pyuria** |  |  |  | |
| - | 1 | 20.9, 15.5-33.8 | 0.0055** | |
| + | 2 | 32.4, 21.4-51.2 |  | |
| **casts** |  |  |  | |
| - | 1 | 25.7, 16.6-41.8 | 0.26 | |
| + | 2 | 29.5, 19.4-62.9 |  | |
| **AI score** |  |  |  | |
| ＜5 | 1 | 22.5, 15.3-33.4 | 0.0026** | |
| ≥5 | 2 | 33.5, 22.1-50.5 |  | |
| **CI score** |  |  |  | |
| ＜4 | 1 | 20.9, 15.4-41.6 | 0.18 | |
| ≥4 | 2 | 28.5, 17.9-40.4 |  | |
| **double track sign** |  |  |  | |
| - | 1 | 24.7, 16.0-45.3 | 0.16 | |
| + | 2 | 31.9, 20.5-53.0 |  | |
| **wireloop sign** |  |  |  | |
| - | 1 | 23.7, 15.7-37.2 | 0.0006*** | |
| + | 2 | 37.2, 23.3-76.7 |  | |
| **endothelial cells hyperplasia** |  |  |  | |
| - | 1 | 22.9, 15.6-33.5 | 0.0005*** | |
| + | 2 | 37.8, 23.7-75.9 |  | |
| **inflammatory cell infiltration** |  |  |  | |
| light | 1 | 22.8, 15.4-39.0 | 0.0011** | |
| heavy | 2 | 34.3, 23.6-53.8 |  | |
| **fibrosis** |  |  |  | |
| - | 1 | 19.2, 15.1-30.0 | 0.013* | |
| + | 2 | 30.8, 19.6-48.7 |  | |

Values are expressed as median and 25–75^th^ percentile. ^a^Mann Whitney U test. SLEDAI-2k, SLE Disease Activity Index 2000; IQR, interquartile range; AI, activity index; CI, chronicity index. *P<0.05; **P<0.01; ***P<0.001.
