# Supplementary figures and images for: Leucine-rich α2-glycoprotein-1 upregulation in plasma and kidney of patients with lupus nephritis
Source: BMC Nephrol. 2020 Apr 6;21:122. doi: 10.1186/s12882-020-01782-0 (PMC7137487; doi:10.1186/s12882-020-01782-0)

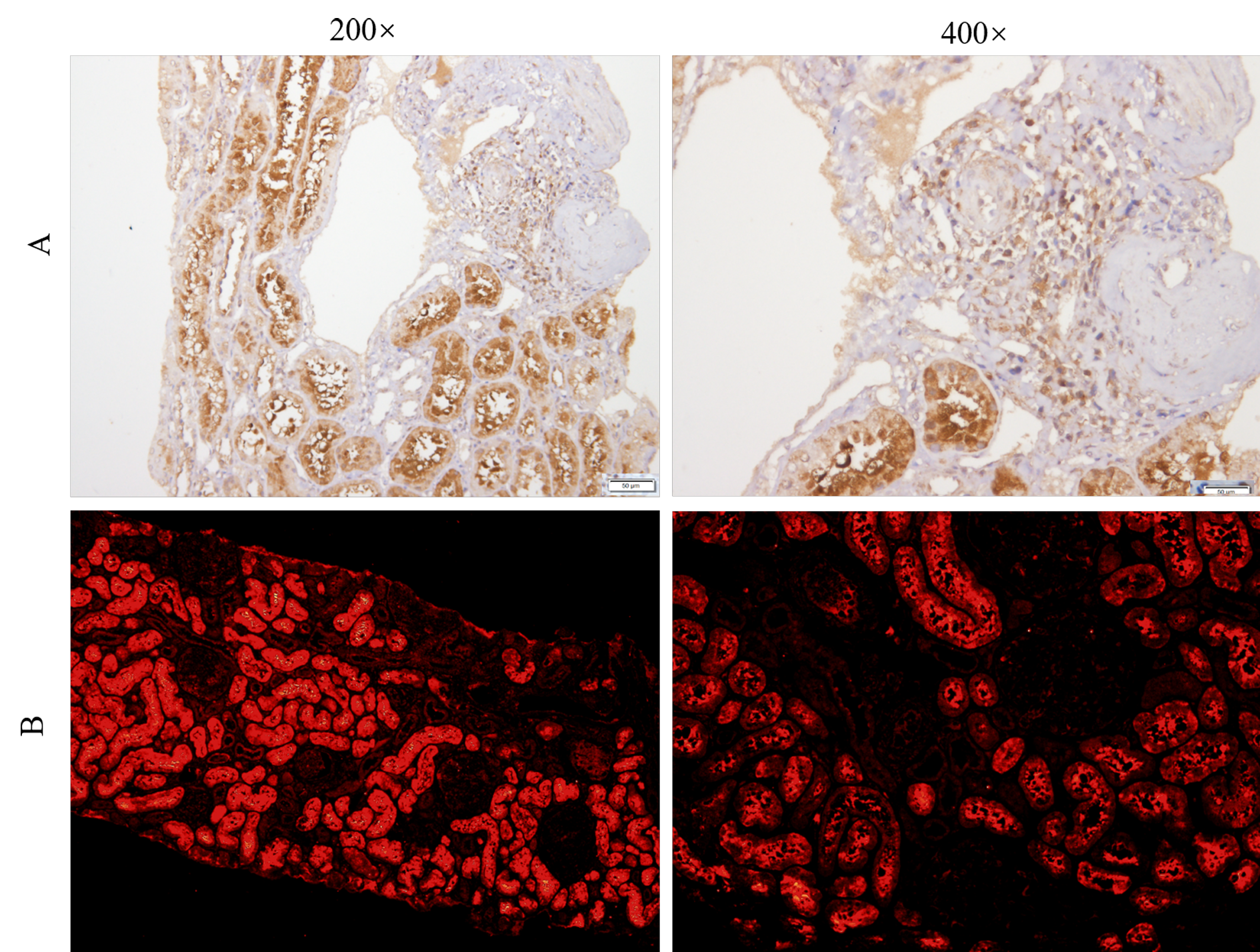

Supplement: Supplementary file 3 — Additional file 3: Figure S1. LRG1 was stained in kidney of LN patients. [file 12882_2020_1782_MOESM3_ESM.tif]
